# Miscellaneous

**Published:** 1880-04

**Authors:** 


### MISCELLANEOUS.

---

8. HOMŒOPATHIC TREATMENT WITH A VENGEANCE.—The *Veterinarian* for November last publishes, without a word of editorial or other comment, the following article, which, from its commendable brevity, we are enabled to reproduce in full: "*Rheumatism, and its successful homœopathic treatment in dogs*, by Samuel Gill, M. R. C. V. S., Hastings. On May 7th I was sent for to go a distance of three miles to see a Scotch Skye terrier, suffering from acute rheumatism. The animal set up a howl directly the hand was passed over the body. Treatment: Tinct. Aconitum, five-drop doses every three hours. May 8th.—Very much improved; ordered Tinct. Belladonna, five-drop doses every three hours. 9th.—Tinct. Rhus, five-drop doses every three hours. June 21st.—A letter was received from the owner containing the following remark: 'Capt. ——— has much pleasure in stating you have been cussessful in the treatment of a Scotch Sye terrier suffering from an acute attack of rheumatism.'" We can, like the *Veterinarian*, forego any comment on this article, but would simply remark, that if the administration of almost fatally large doses constitutes Homœopathy, then we are really uncertain as to what our own status is.

---

9. Disturbance in a Continental Veterinary School.—In his excellent address before the class of the Royal College of Veterinary Surgeons, Professor Cobbold took occasion to rebuke the students of the college for some disturbance they had made, and referring to the school at Alfort, which he had recently visited, stated, "At Alfort the discipline is exceedingly strict. When I privately mentioned that rude interruptions were occasionally witnessed at the Royal Veterinary College, I was given to understand that such conduct would not for an instant be tolerated at the great republican establishment." The distinguished helminthologist has, however, received a rather inopportune *dementi*, especially as regards the alleged sobriety of French character. The *Progrès Medicale*, of the 6th of December last, contains an item to the effect that the students had protested against the diet and other regulations made at the Alfort school, and had raised quite a rumpus, destroying the furniture of the dormitories and the refectory. A dozen of the most tumultuous members of the class were expelled. There is nothing like spirit for young people; and while, on the one hand, we consider rude Britannic interruptions of a lecturer as unmanly, and on the other, Gallic demonstrations of fury vented on articles of furniture as childish, yet there are occasions on which to expect the students to be the passive recipient either of bad lectures or school-boy regulations would be an insult to their intelligence. If a lecturer on obstetrics, derived from the human medical profession, for example, on attempting to demonstrate the relations of the pelvic diameters, places the sacrum wrong-end forwards, between the *ilia*, and on observing his error proposes to thrust the coccygeal end through the obturator foramen, and to illustrate the presentations and positions by means of a four-inch toy horse, or finally, when endeavoring to write out on the blackboard the component parts of the ovum, chalks down the word "Tunica," and then, at an apparent loss, accepts the suggestion, "Ruyshiana" from a member of the class, who ventures this word in random fun, we are inclined to the belief that even the most loyal and quiet student may find the demands of regulating discipline overtaxing. The school should not be an anarchical institution, it is true; but as little should it become a despotism. The right to criticize their superiors will always be a privilege of students, and he is the wisest teacher who, within reasonable limits, devotes some attention to the requirements and capacity of his class, and does not attempt to override them, booted and spurred, in such a manner that nothing but the mechanical and mind-killing "cram-quizz" can enable them to make even a show of following up his subject.

10. INFLUENCE OF TAXATION ON THE CANINE POPULATION.—From the year 1867 to 1878, inclusive, the number of dogs in the Grand Duchy of Baden sank from 39,879 to 25,094, in consequence of the dog tax.—*Bad. Thieraztliche Mittheilungen, Jan. 1880.*

---

11. COLTS POISONED BY FERMENTED RYE.—M Bastin was called to a farmer, who reported that two of his colts had died during the night, and that eleven others were seriously ill. He arrived in time to see a third one perish. One animal exhibited violent intestinal pain—anxious expression ; another was affected with a profuse and fetid diarrhœa; all were anxious in expression. It seemed that the more vigorous animals were much more prostrated than the weaker. It was found, on inquiry, that a laborer had mixed rye with water, and left portions of it so, adding a little every day to make up for what had been eaten, so that the water and rye had probably been fermenting four days; the rye accordingly manifested a penetrating odor. There being no pharmacy in the neighborhood, M. Bastin gave each animal half a liter of coffee and cold affusions, a bolus of nux vomica on account of the prostration (some of the animals were even paralysed), an aloetic purgative, and, instead of water, a linseed decoction. In all, five colts died; the others recovered after a prolonged illness, showing also suppression of urine. On the autopsy nothing was noticed in the thoracic cavity of moment; there was a penetrating odor noticeable on opening the stomach, due to the fermenting rye with which this organ was filled; there was gastro-enteric inflammation. The central nervous centres were congested. Professor Lorge, to whom the stomachs and their contents were sent for analyses, stated that death was due to poison, not by the alcohol resulting from the fermentation, but by the presumable formation of some organic poison.—*Annales de Medecine Vétèrinaire, Janvier, 1880.*

---

12. A horse was twice brought to the Munich clinic, which, though suffering evident thirst and manifesting an eager desire for water, was unable to take a drop of water for *fifteen days.* The horse was lost sight of. In the absence of any other assignable cause, a cerebral affection was supposed.—*Yahresbericht d. K. C. Thierarzneischule in Munchen, 1879.*

---

13. DANGER OF LARYNGEAL INSUFFLATION IN HORSES.—In one of three horses dying from puenomina due to foreign bodies, the cause was found to have been the administration of a powder for the larynx.—*Ibidem.*

---

14. The *Veterinarian* quotes a German periodical to the effect that Gen. Voights-Reetz introduced fleshmeal into the food of the horses of a cavalry regiment. The condition and appetite improved considerably. Dunkelberg, who made the suggestion, states that for each kilogram of dried fleshmeal 5.25 grams potassium chloride, 27.9 potassium phosphate, and 2.9 grams of magnesium phosphate should be administered.

15. A Hairless Horse.—An aid-de-camp of General Kaufmann, Governor General of Turkestan, presented a hairless horse to the Zoölogical Gardens of Moscow. The exact history of the origin of this rare variety is shrouded in obscurity. It is hardly probable that this animal is a specimen of a peculiar Tartar breed of horses, and is more likely an individual peculiarity, such as that noted in the white elephant, the "wooly horse," *et cetera*. The animal has a beautiful shape, and its skin, which is of a vivid reddish tinge, does not present the slightest indication of hair. This circumstance renders the animal very susceptible to the weather, and it has to be kept covered with blankets.—*From Gazette de St. Petersbourg per L'Union medicale.* There is no special mention as to whether the mane and tail hairs are also absent.—*Editorial Note.*

---

16. A New Veterinary Society.—Seventeen veterinarians, residing in and around the cities of New York and Booklyn, met on the 13th of February to organize an association for the purpose of promoting the discussion of veterinary scientific topics and the elevation of the veterinary professional standard. The name selected for the society is the Academy of Comparative Medicine and Surgery. Time of meeting, the second Tuesday in each month. The vote for officers resulted in the unanimous election of Dr. Allan S. Heath as President; Dr. Platt was elected Recording Secretary; Dr. Bers as Treasurer.

---

17. From a letter by Dr. Bowler, V. S., of Cincinnati, we glean the following: "The castrating écraiseur which Farmer Miles has exhibited to the English veterinarians was first suggested to me by Dr. R. Jennings of Philadelphia, but now of Detroit, Mich. This was seventeen years ago, at which time we made a drawing of the instrument now used, and of which we had two made by a surgical instrument maker in Philadelphia.

"At the first meeting of theUnited States Veterinary Medical Association, held a the Astor House, in New York City, Dr. Jennings introduced this instrument to the members, and spoke of the advantages derived from its use in castrating. . . . . . . . . . . . . . . . . . But from that date to the present, I, and also Dr. Jennings, have continued to use it, and have, during all these years, castrated thousands of animals of all ages, without an accident or the loss of a single one; and *it must be borne in mind that during this period a considerable number of these animals were cryptorchus.* About ten or twe've years ago I had a number of these instruments manufactured, and sold them to parties in various parts of the States. *Ten years ago* I furnished a gentleman friend of mine with one of the instruments, in order that he might castrate his own stock, he being a large stock raiser and owner of a large farm in Illinois. He has used it up to the present time, castrating many thousand head of stock during that time and, as he informed me a short time ago, that he had never lost a single horse. . . . . . . . . . . . . . During my last visit to Europe, in 1873, I called on Professors Axe and Pritchard, and endeavored to explain to them the advantages to be derived from the use of the castrating ecraiseur, and I must say I was rather surprised when they informed me that they still continued to adopt the oldfashioned method of castrating. I also remember explaining to Professor Axe my methods of throwing and fastening horses, without any risk or accident,* which I have no doubt he will at once recall to mind when he sees this article. And now, Messrs. Editors, I must ask your correspondents to give us something new in the future other than reports of colic cases which yield to Sp. Nit. Eth.and Tr. Opii. They are getting very stale. And then there is that *dog case*, and as the writer states, treated homœopathically. Five-drop doses of the saturated tincture of aconite to a little Scotch terrier, every three hours, and to be followed by the same dose of belladonna.

"If he calls this homœopathy, what, in Heaven's name, are his allopathic doses? I should hate to be that man's dog. I wonder how much whiskey he puts in his water at one time? I should much like to know, as I feel satisfied he uses allopathic doses on those occasions."—*The Veterinarian*, *January*, *1880*

---

18. "HOMŒOPATHIC" REPLY.— Under the heading "Homœopathic treatmen of Disease. Reply from Mr. S. Gill, M. R. C. V. S., Hastings, to Dr. Bowler, V.S., Cincinnati, Ohio," the M. R. C. V. S. (*!sic*) publishes an incoherent lucubration, somewhat lengthier than the remarkable article which occasioned the dispute, and which will be found reproduced in full elsewhere (Miscellaneous). The specially interesting part of the letter is this: "Gentlemen—In January month's issue of the *Veterinarian* I find a letter, principally on castration, by a Dr. Bowler, styling himself also a veterinary surgeon, although I do not find his name in our college register, in which he attacks my system of cure by homœopathy in such a manner that I must, by your kind permission, repeat that the dog *was cured*, and as such it might be asked, What need is there of further testimony? . . I am (unfortunately for the doctor's notions of my habit) a *total abstainer*, therefore do not require either 'allopathic' or homœopathic doses of whiskey and water, which he, as a medical man, should know is poison to the human frame, and destructive of tissue. Besides which, it and such like stimulants often lead men blindly on to speak glibly, 'in Heaven's name', of persons and things they know nothing about."

We fear that Dr. Bowler, V. S., was in error in his diagnosis. The mental defect noticable in the above letter, and in the article referred to, is altogether of such a character that it cannot be explained on the strength of an acute delirium following excessive doses of alcohol! In such delirium, with a great deal of incoherence and absurdity, there is still a slight glimmering of the light of human reason, such as is conspicuously absent in the M. R. C. V.'s writings. No; it is a chronic, incurable, and probably congenital defect of the cerebral centres. While the unfortunate sufferer from this sad defect merits our deep commisseration, we cannot suppress the suggestion that his case hardly constitutes an argument in favor of "total abstinence," though it is difficult to decide whether, in this case, imbecility is due to the total abstinence, or the total abstinence to the imbecility.

---

* Taught by James Hamill, D. V. S., lecturing on shoeing at the Veterinary College for the lats two years.

